# Maternal Vitamin D Status, Oxidative Stress, and Implications for Neonatal Development: A Cross-Sectional Study

**DOI:** 10.3390/metabo16010019

**Published:** 2025-12-24

**Authors:** Tania Flores-Bazán, Jacqueline Scarlett Barreto-González, José Pedraza-Chaverri, Omar Noel Medina-Campos, Araceli Castañeda-Ovando, Jeannett Alejandra Izquierdo-Vega, Diego Estrada-Luna, Martha Eunice Rodríguez-Arellano, Angélica Saraí Jiménez-Osorio

**Affiliations:** 1 Área Académica de Enfermería, Instituto de Ciencias de la Salud, Universidad Autónoma del Estado Hidalgo, Circuito Ex Hacienda La Concepción S/N, Carretera Pachuca-Actopan, San Agustín Tlaxiaca 42160, Mexico; fl467888@uaeh.edu.mx (T.F.-B.); destrada_luna@uaeh.edu.mx (D.E.-L.); 2 Servicio de Ginecología y Obstetricia, Hospital Regional “Lic. Adolfo López Mateos”, Instituto de Seguridad y Servicios Sociales de los Trabajadores del Estado (ISSSTE), Ciudad de México 01030, Mexico; dra.scarlettbarretog@gmail.com; 3 Departamento de Biología, Facultad de Química, Universidad Nacional Autónoma de México, Ciudad de México 04510, Mexico; pedraza@unam.mx (J.P.-C.); omarnoelmedina@gmail.com (O.N.M.-C.); 4 Área Académica de Química, Instituto de Ciencias Básicas e Ingeniería, Universidad Autónoma del Estado de Hidalgo, Carretera Pachuca, Tulancingo 42039, Mexico; ovandoa@uaeh.edu.mx; 5 Área Académica de Medicina, Instituto de Ciencias de la Salud, Universidad Autónoma del Estado de Hidalgo, Circuito Ex-Hacienda de la Concepción S/N, Carretera Pachuca-Actopan, San Agustín Tlaxiaca 42160, Mexico; ivega@uaeh.edu.mx; 6 Laboratorio de Medicina Genómica, Hospital Regional “Lic. Adolfo López Mateos”, Instituto de Seguridad y Servicios Sociales de los Trabajadores del Estado, Ciudad de México 01030, Mexico; marthaeunicer@gmail.com

**Keywords:** pregnancy, oxidative stress, neonatal outcomes, 25-hydroxyvitamin D, vitamin D insufficiency

## Abstract

**Background**: Vitamin D (VD) plays a central role in calcium homeostasis during pregnancy and has been implicated in redox-related biological processes. While VD deficiency (VDD) has been consistently associated with adverse pregnancy outcomes, the relationships between VD insufficiency (VDI), maternal antioxidant-related biomarkers, and neonatal outcomes remain incompletely characterized, particularly during the third trimester. **Objective**: To determines the prevalence of VDI in third-trimester pregnant women and to examine its associations with antioxidant-related markers and selected neonatal outcomes. **Methods**: A cross-sectional study was conducted among pregnant women in the third trimester attending a tertiary referral hospital in Mexico City. Maternal serum 25-hydroxyvitamin D (25-OHD) concentrations were measured, along with a panel of redox-related markers, including 2,2-diphenyl-2-2picrylhydrazyl (DPPH) radical scavenging activity, reduced glutathione (GSH), glutathione S-transferase (GST), glutathione peroxidase (GPx), and oxygen radical absorbance capacity (ORAC). Neonatal anthropometric parameters were recorded at birth. Associations between maternal VD status, redox-related markers, environmental factors, and neonatal outcomes were evaluated using appropriate statistical analyses. **Results**: A high prevalence of VDI was observed in the study population. Maternal VDI was associated with lower activities of GSH, GST, and GPx. Passive exposure to tobacco smoke and season of sampling were also associated with lower VD concentrations. Neonates born to women with VDI had higher birth weight compared with those born to women with sufficient VD concentrations. Maternal serum 25-OHD concentrations correlated positively with selected antioxidant enzyme activities. **Conclusions**: In this cohort of third-trimester pregnant women, VDI co-occurred with environmental factors, differences in maternal redox-related markers, and selected neonatal outcomes. These findings support an associative framework in which suboptimal VD status during the third trimester is accompanied by variations in redox-related markers. Longitudinal and mechanistic studies are needed to clarify the temporal sequence and biological relevance of these associations.

## 1. Introduction

Pregnancy constitutes a critical period characterized by substantial metabolic and nutritional demands, particularly during the second and third trimesters. Vitamin D (VD), a secosteroid hormone, is an essential micronutrient with a central role in calcium homeostasis and fetal skeletal mineralization, a process that reaches its peak during the third trimester. Adequate maternal nutrient status is therefore fundamental, as it is a well-established determinant of pregnancy outcomes, neonatal health, and long-term disease risk in the offspring [1,2].

Assessment of VD status is based on serum concentrations of 25-hydroxyvitamin D (25-OHD); however, considerable debate persists among major health organizations, including the Institute of Medicine (IOM) and the Endocrine Society (ES), regarding optimal diagnostic thresholds for sufficiency during pregnancy, commonly defined as ≥30 ng/mL [3]. While VD deficiency (VDD) has been consistently associated with adverse outcomes such as preeclampsia [4,5], gestational diabetes mellitus (GDM) [6,7], preterm birth, and low birth weight [8,9,10], the clinical implications of VD insufficiency (VDI) remain less clearly characterized. Nevertheless, accumulating evidence suggests that VDI has been associated with pregnancy complications [11], including an increased risk of preterm birth [12], reduced fetal bone growth and shorter gestation [13], and a higher risk of insulin resistance [14], albeit with a generally lower risk profile than that reported for VDD [15,16,17].

Epidemiological data indicate that the prevalence of VDD during pregnancy ranges widely—from 5% to 90%—largely influenced by geographic latitude, environmental exposure, and socioeconomic factors [18]. In developed countries, VDD prevalence has been estimated at approximately 24% in the United States, 37% in Canada, and 40% in continental Europe, whereas rates exceeding 80% have been reported in countries such as Pakistan, India, and Bangladesh [19]. A recent systematic review reported that 54% of pregnant women have serum 25-OHD concentrations below 50 nmol/L (20 ng/mL) [20], underscoring hypovitaminosis D as a major global public health concern. In contrast, VDI during pregnancy has received comparatively less attention, despite its high prevalence and potential clinical relevance.

Interpretation of VD status is further complicated by the multitude of factors influencing serum 25-OHD concentrations, including seasonal variation, ultraviolet B (UVB) exposure, skin pigmentation, body mass index (BMI), and dietary habits [21,22]. Emerging evidence also suggests that VD may be linked to antioxidant and immunomodulatory pathways during pregnancy. At sufficient levels, its active form, calcitriol, has been associated with the regulation of antioxidant enzymes and inflammatory mediators, as well as with lower concentrations of oxidative damage markers in experimental and observational studies [23,24].

Concurrently, normal pregnancy is characterized by a physiologically heightened oxidative state driven by increased basal metabolic rate, augmented placental mitochondrial activity, and increased insulin resistance in the last trimester [25]. Reduced glutathione (GSH) is regarded as the primary intracellular antioxidant and acts as a sensitive indication of cellular redox equilibrium; diminished levels in the placenta have been linked to pregnancy issues such as preeclampsia [26,27]. Its associated enzymes–glutathione peroxidase (GPx), which participates in the detoxification of hydrogen peroxides, and glutathione S-transferase (GST), involved in the conjugation of reactive electrophiles–are commonly used to characterize the glutathione-dependent antioxidant system in pregnancy-related research [28]. In addition, the radical 2,2-diphenyl-2-2picrylhydrazyl (DPPH) radical scavenging assay and the oxygen radical absorbance capacity (ORAC) assays are included as experimental, non-clinical indicators of total antioxidant capacity, capturing plasma ability to neutralize free radicals through electron- and hydrogen-transfer mechanisms [29]. These assays do not represent validated clinical markers and lack pregnancy-specific reference ranges; therefore, they are interpreted herein as relative, group-level measures that complement enzymatic antioxidant markers rather than as indicators of clinical antioxidant status.

Environmental and lifestyle factors may further exacerbate oxidative stress (OS) and have been associated with placental dysfunction and adverse pregnancy outcomes. A systematic review of VD supplementation during pregnancy reported significant reductions in malondialdehyde (MDA) levels and increases in GSH concentrations, suggesting a potential association with enhanced antioxidant defense [30]. Similarly, optimal VD concentrations have been correlated with higher circulating GSH concentrations in observational settings [31].

Despite this growing body of evidence, important knowledge gaps persist. Most studies have focused on overt deficiency rather than insufficiency; few have simultaneously assessed enzymatic and total antioxidant capacity markers; and data from Latin America populations–where environmental, nutritional, and sociodemographic factors may differentially influence VD status and redox balance–remain limited. Moreover, the interplay between suboptimal VD concentrations and OS in relation to maternal and neonatal outcomes during the third trimester remains poorly understood.

Study rationale and objectives. While individual associations between VDI and OS, as well as between OS and adverse pregnancy outcomes, have been previously reported, their simultaneous evaluation during the third trimester has been limited. This gap is particularly evident in Latin American populations. Therefore, the central research question of this cross-sectional study is whether maternal VDI during the third trimester is associated with alterations in antioxidant markers and with differences in selected neonatal outcomes.

Based on existing evidence suggesting associations between suboptimal VD status and impaired antioxidant defenses, we hypothesized that lower maternal 25-OHD concentrations (<30 ng/mL) are associated with a less favorable antioxidant profile and with variations in neonatal outcomes, without implying causal or mechanistic relationships. Accordingly, the aims of this study are as follows: (1) determine the prevalence of VDI in a cohort of third trimester pregnant women; (2) compare enzymatic and non-enzymatic OS markers (DPPH, GSH, GST, GPx, and ORAC) between women with sufficient and insufficient VD status; (3) examine associations between maternal VD status, OS markers, and selected neonatal outcomes; and (4) evaluate the combined associative and predictive contribution of VD status and OS markers in a tertiary referral hospital in Mexico City.

## 2. Materials and Methods

### 2.1. Study Design and Participants

This observational, cross-sectional study was conducted between April 2024 and May 2025 at the Regional Hospital ‘Lic. Adolfo López Mateos’ in Mexico City, Mexico. Participants were recruited during routine third-trimester prenatal care visits.

Inclusion criteria were: women in the third trimester of gestation (28–32 weeks); clinically healthy or scheduled for an iterative cesarean section; continuous residence in Mexico City for at least five years; affiliation with the Institute for Social Security and Services for State Workers (ISSSTE); receipt of prenatal care at the study site; and provision of written informed consent.

Exclusion criteria included pre-existing chronic conditions prior to pregnancy (pregestational diabetes mellitus, systemic arterial hypertension, cancer, or autoimmune diseases); a history of stillbirths or high-risk pregnancy; requirement for specialized monitoring in a maternal–fetal medicine unit; or refusal to provide informed consent.

### 2.2. Sample Size Calculation

Sample size was calculated a priori to estimate the prevalence of VDI in the target population, using a finite population correction. The following parameters were applied: total eligible population during the study period (N = 150), expected prevalence (*p* = 0.30), margin of error (*d* = 0.05), and a 95% confidence level (Z = 1.96). This calculation yielded a minimum required sample size of 85 participants.

To account for potential non-response or incomplete data, a recruitment target of 120 pregnant women was established, based on historical hospital records, recruitment feasibility was estimated within the planned period. Although the initial recruitment target was defined for a six-month window, enrollment was extended to ensure attainment of the minimum sample size and adequately represent seasonal variability.

It should be noted that this sample size calculation was designed exclusively for prevalence estimation. The study was not powered a priori to detect differences between-group differences or to support multivariable regression analyses. Accordingly, all comparative and multivariate analyses were conducted for exploratory and associative purposes, and no claims regarding statistical power for these analyses are made.

### 2.3. Ethical Considerations

This study was conducted in accordance with the ethical principles of the Declaration of Helsinki [32], and was approved by the Research Ethics Committee of the ISSSTE ‘Lic. Adolfo López Mateos’ (Protocol No. 323.2024).

Prior to enrollment, all participants received comprehensive verbal and written information regarding the study’s objectives, procedures, potential risks, and benefits. Written informed consent was obtained from each participant.

Clinical and biochemical data were extracted from medical records corresponding to routine third-trimester prenatal care, following informed consent. Blood samples were collected exclusively for the determination of VD concentrations. Confidentiality and data anonymity were rigorously maintained throughout data collection, storage, and analysis.

### 2.4. Data Collection and Variables

Sociodemographic and clinical data were obtained using structured questionnaires and medical record reviews. Detailed operational definitions for all exposure, outcome, and potential confounding variables are provided in Supplementary Material Table S1.

Collected variables included the following:

Maternal characteristics: age, pre-pregnancy body mass index (BMI), gestational weight gain (derived from medical records), education level, occupation, VD supplementation status, and smoking exposure.

Clinical obstetric data (from medical records): gestational age at delivery (confirmed by first-trimester ultrasound), parity (nulliparous/multiparous), mode of delivery (vaginal/cesarean), and pregnancy complications including gestational diabetes and preeclampsia (diagnostic criteria detailed in Supplementary Material Table S1).

Neonatal outcomes (from medical records): gestational age at birth assessed by the Capurro score, Apgar scores at 1 and 5 min, head circumference, mid-upper arm circumference, birth weight, and neonatal intensive care unit admission status with documented reasons.

Biochemical parameters (from medical records): serum uric acid, total cholesterol, low-density lipoprotein cholesterol (LDL-C), high-density lipoprotein cholesterol (HDL-C), triglycerides, hemoglobin, and glucose levels, when available from routine third-trimester.

Data were managed using a custom-designed Microsoft Excel database with locked cells and predefined values ranges to minimize entry errors. Double data entry was performed independently by two researchers. Discrepancies were identified through automated cross-validation and resolved by consensus with senior investigators. A random 10% of records was verified against source documents. Variables with >5% missing data were pre-specified for exclusion from multivariate analyses.

#### 2.4.1. Construction of the Occupational Sun Exposure Variable

Occupational sun exposure was derived from self-reported occupation and classified by consensus among three independent researchers according to predefined criteria:

High exposure: Occupations involving >75% of the workday outdoors.
Medium exposure: Occupations involving 25–75% of the workday outdoors, or frequent intermittent exposure.
Low exposure: Occupations involving <25% of the workday outdoors.
For example, the legal profession was classified as “low exposure” because core job tasks are predominantly performed indoors. Sun exposure during commuting was considered incidental and not part of occupational exposure.

#### 2.4.2. Assessment of Seasonal Variation

Season at blood sampling was categorized using astronomical dates for the collection year (Supplementary Material Table S1, page 2):

Spring: March 20–June 20
Summer: June 21–September 21
Autumn: September 22–December 20
Winter: December 21–March 19
Season was included a priori as a potential confounder of serum 25-OHD concentrations.

#### 2.4.3. Definition of VD Supplementation Status

VD supplementation was assessed through structured interviews and verified when available, using prescription records or supplement containers. Most users report initiating prenatal multivitamins containing cholecalciferol (400–600 IU) during the first trimester.

Supplementation was defined as regular intake (≥3 times per week) of any supplement containing ≥400 UI of VD (cholecalciferol or ergocalciferol) for at least one month prior to blood sampling. Participants were classified accordingly as “yes” or “no.”

### 2.5. Vitamin D Analysis

Peripheral blood samples were collected from fasting participants during the third trimester of pregnancy (28–32 weeks of gestation) using standardized venipuncture procedures. Blood was drawn into one 6 mL clot activator tube for serum and one 6 mL ethylenediaminetetraacetic acid (EDTA) tube for plasma.

Participants were instructed to attend the sampling visit after an overnight fast of at least 8 h. Sample collection was performed by trained nursing staff in accordance with institutional biosafety and quality-control protocols.

Immediately after collection, samples were protected from light and processed within 30 min of collection to minimize degradation and oxidative alterations. Samples were centrifuged at 3500× *g*, 10 min, 4 °C, aliquoted in triplicate (500 µL), and stored at −80 °C until analysis. Aliquoting was performed to avoid repeat freeze–thaw cycles, and no sample underwent more than one freeze–thaw cycle prior to biochemical determinations.

All biochemical determinations, including serum 25-OHD and OS-related markers, were performed in duplicate, and the mean value of the two measurements was used for statistical analyses. When the coefficient of variation between duplicates exceeded predefined acceptable limits, the assay was repeated.

Serum 25-OHD concentrations were quantified externally at an (Biogenetics and other Vector’s diseases lab^®^, Hidalgo, México) using liquid chromatography-tandem mass spectrometry (LC-MS/MS). Analytical procedures included batch-randomized duplicate samples, NIST-traceable calibration standards, and internal quality controls. The inter-assay and intra-assay coefficients of variation were <5% and <3%, respectively, ensuring high analytical precision.

VD status was classified using thresholds commonly applied in observational studies of pregnant women. VDI was defined as 25-OHD concentrations between 21 and 29 ng/mL, and sufficiency as concentrations ≥30 ng/mL [33,34,35]. These cutoffs are frequently employed in studies examining non-skeletal outcomes during pregnancy.

Although alternative thresholds proposed by the IOM (≥20 ng/mL) are primarily derived from skeletal outcomes in the general population, the ≥30 ng/mL cutoff was selected a priori to ensure comparability with the pregnancy-focused literature and to align with the exploratory and associative aims of the present study.

### 2.6. Assessment of Oxidative Stress and Antioxidant Status

A panel of enzymatic and non-enzymatic assays was performed on plasma and/or erythrocyte lysates to characterize OS-related antioxidant status. Currently, there are no universally validated OS or antioxidant markers with established pregnancy-specific reference ranges or diagnostic cut-off values. Consequently, no single marker can be considered a gold standard for assessing OS during pregnancy.

In this context, the present study employed a panel of antioxidant-related markers that have been previously used in pregnant women [36] to describe redox-related activity. These markers were selected to capture complementary aspects of antioxidant defense, rather than to diagnose OS or to compare absolute values against clinical reference standards.

All OS-related markers were therefore analyzed and interpreted as relative continuous variables for within-cohort comparisons and associative analyses, acknowledging the absence of validated pregnancy-specific thresholds.

Plasma and erythrocyte fractions were stored at −80 °C and thawed only once prior to analysis.

(a) DPPH Radical Scavenging Assay

The free radical-neutralizing capacity was evaluated using the DPPH assay, based on the method described by Koren et al. [37], with minor modifications. This assay provides an index of non-enzymatic antioxidant capacity under controlled in vitro conditions. Briefly, 37.6 µL of the sample was mixed with 20 µL of 2 mM DPPH solution (Sigma-Aldrich, St. Louis, MO, USA). After 2 min of incubation at room temperature, 400 µL of ethanol (JT Baker, Xalostoc, Mexico) was added. The mixture was vortexed and centrifugated at 3000× *g* for 3 min. A total of 370 µL of supernatant was transferred to a 96-well microplate (Costar, cat. #9017), and the absorbance was measured at 517 nm using a Synergy HT1 multimode microplate reader (Biotek Instruments Inc., Winooski, VT, USA).

The percentage of DPPH radical scavenging activity was calculated using the following equation, where C0 represents the absorbance of the control (water, DPPH, and ethanol without sample) and SX the absorbance of the sample:

% DPPH scavenging = [(C0 − SX)/SX] × 100)

(b) Oxygen Radical Absorbance Capacity (ORAC) Assay

Antioxidant activity was quantified using the ORAC assay, following Huang et al. [38] with adaptations. ORAC values were used as an integrated measure of peroxyl radical scavenging capacity and are reported to comparative purposes within the study population.

Briefly, 25 µL of plasma, Trolox standard, or blank was mixed with 150 µL of fluorescein (50 nM) in a 96-well plate (Costar, cat. #3915). The reaction was initiated by adding 25 µL of 2,2’-Azobis(2-methylpropionamidine) dihydrochloride (AAPH). The microplate was immediately placed in a preheated (37 °C) Synergy HT microplate reader, and fluorescence (excitation = 485 nm, emission = 520 nm) was measured every minute for 90 min.

ORAC values were calculated based on the area under the fluorescence decay curve (AUC) and expressed as micromoles of Trolox equivalents per liter (µM TE).

(c) Glutathione (GSH) levels

The cellular redox status was evaluated by measuring GSH levels using an enzymatic recycling assay based on the method of Rahman et al. [39]. GSH levels were interpreted as descriptive indicators of antioxidant-related biochemical status.

Sample preparation:

200 µL of plasma was mixed with 100 µL of 0.6% sulfosalicylic acid (Sigma-Aldrich, St. Louis, MO, USA) to precipitate proteins. The mixture was centrifuged at 8000× *g* for 10 min, and the supernatant was collected and kept on ice until analysis.

Assay procedure:

60 µL of the prepared supernatant was transferred to a well, followed by 120 µL of Ellman’s reagent (5,5’-dithiobis-(2-nitrobenzoic acid), (DTNB), and incubated for 30 s. Then 50 µL of 0.8 mM NADPH were added to each well. The absorbance was read at 412 nm every minute for 3 min.

GSH concentration was determined by interpolation from a standard curve (0–26.4 nmol/L) prepared in 0.1 M potassium phosphate buffer (pH 7.5) containing 5 mM EDTA. All reagents were prepared fresh and protected from light.

(d) Glutathione peroxidase (GPx) activity

The enzymatic activity of GPx was determined based on the protocol established by Lawrence and Burk [40]. GPx activity was assessed to describe enzymatic antioxidant-related activity.

The reaction mixture (final volume of 0.35 mL) contained 1 mM EDTA, 1 mM NaN_3_, 0.2 mM, Nicotinamide Adenine Dinucleotide Phosphate (NADPH), 1 U/mL glutathione reductase, and 1 mm glutathione in 50 mM potassium phosphate buffer (pH 7.0). The reaction was initiated by adding 2.5 mM H_2_O_2_.

The decrease in absorbance at 340 nm, corresponding to NADPH consumption, was recorded for 3 min. GPx activity was derived from the linear rate of absorbance change. One unit of activity (U) is defined as the amount of enzyme that catalyzes the oxidation of µmol of NADPH per minute under the assay conditions. Specific activity is expressed as units per milliliter of sample (U/mL).

(e) Glutathione-S-Transferase (GST) activity

GST enzymatic activity was assayed based on the method of Habig et al. [41]. GST activity was included as a descriptive marker of phase II antioxidant-related enzymatic activity.

The reaction was carried out in a 0.05 M potassium phosphate buffer (pH 6.5) containing 2 mM GSH and 1 mM 1-chloro-2,4-dinitrobenzene (CDNB) as substrates. The increase in absorbance at 340 nm, resulting from the formation of the CDNB-GSH conjugate, was calculated and expressed in units per milliliter (U/mL), with one unit (U) corresponding to the conjugation of 1 µmol of CDNB to GSH per minute under the specified conditions.

### 2.7. Statistical Analysis

Statistical analyses were performed using IBM SPSS Statistics [version 26]. Normality of continuous variables was assessed using the Kolmogorov–Smirnov test. Continuous variables are presented as mean ± standard deviation or median (interquartile range). Categorical variables are expressed as frequencies and percentages.

Group comparisons between VD sufficiency and insufficiency were performed using Student’s t-test for normally distributed continuous variables and the Mann–Whitney U test for non-normally distributed variables. Categorical variables were compared using the Chi-square test or Fisher’s exact test as appropriate. Correlation analyses were conducted to assess the direction and strength of associations between VD concentrations and variables showing statistical significance.

The stepwise approach was used to conduct multivariate linear regression analysis with VD levels as the dependent variable. After controlling for age and BMI, the first model was used to determine the predictive capacity of redox markers (DPPH, ORAC, GPX, GST, and GPX) on VD variability. In order to identify factors with the best ability to predict VD value fluctuation, the second model included variables that were significant in the univariate analysis (GPx, GST, GSH, infant weight, Capurro index, mother’s smoking status, and collection season). The Variance Inflation Factor (VIF) analysis was used to identify multicollinearity, and variables with values greater than five were eliminated. The distribution of the residuals was ascertained using the Shapiro–Wilk test, symmetry, and kurtosis analysis using STATA v.16 software. White test was used to assess homoskedasticity and if the Durbin–Watson statistic (between 1–3) was used to estimate autocorrelation. The models satisfied the requirements of homoskedasticity, non-multicollinearity, autocorrelation, and residual normality (Supplementary Table S2).

## 3. Results

### 3.1. Participant Flow and Baseline Characteristics

**Figure 1:**
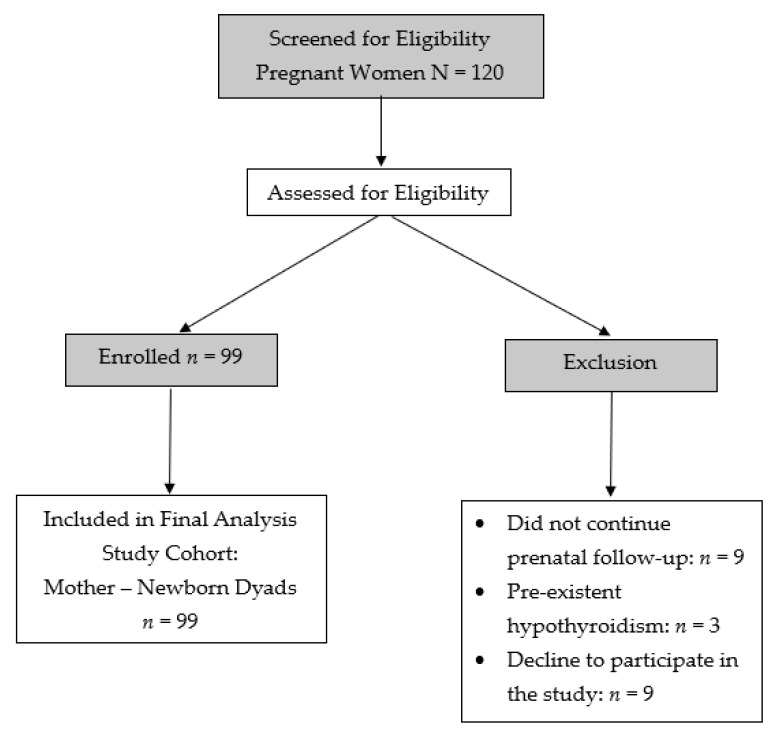
Flowchart of participant recruitment and inclusion in the final analytical cohort (*n* = 99 mother–newborn dyads).

A total of 120 pregnant women in their third trimester were assessed for eligibility, all of whom met the inclusion criteria and were initially enrolled. After enrollment, 21 participants were excluded from the final analysis due to loss to prenatal follow-up (*n* = 9), pre-existing hypothyroidism (*n* = 3), or withdrawal of consent (*n* = 9). The final analytical cohort therefore comprised 99 mother–newborn dyads (Figure 1). The mean maternal age was 29.96 ± 4.54 years (range: 16–39 years).

### 3.2. Association Between VD Status and Antioxidant/Redox Markers

**Figure 2:**
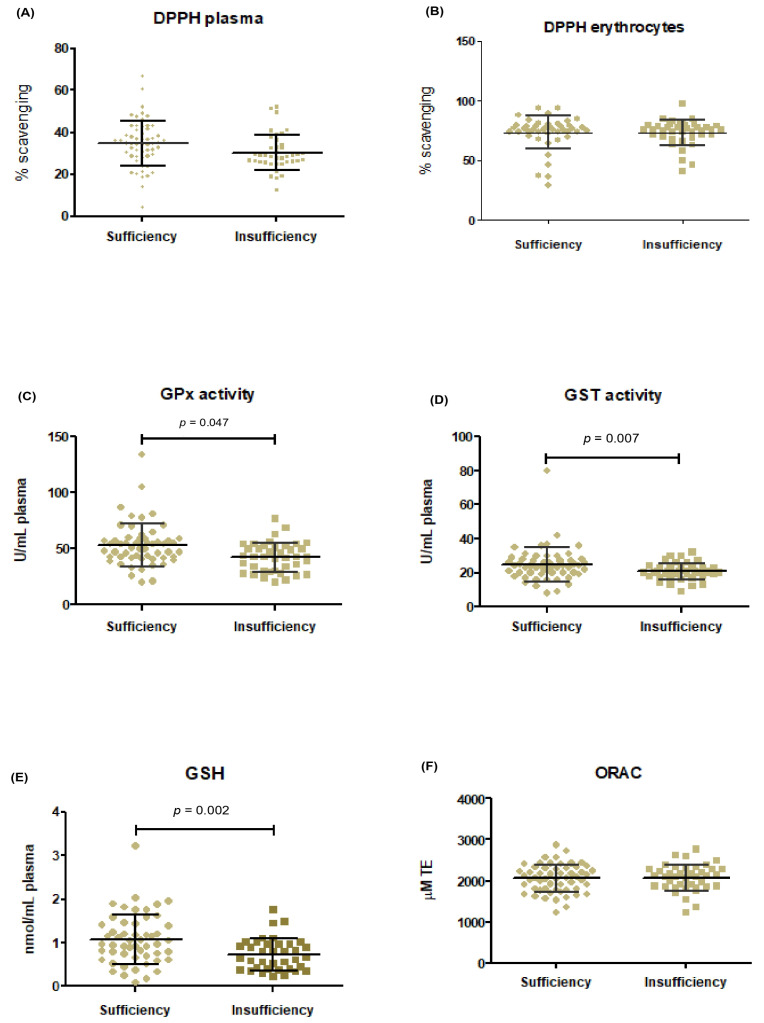
Comparison of antioxidant and redox-related markers by maternal VD status, Panels (**A**–**E**) show plasma DPPH scavenging activity, GPx activity, GST activity and GSH concentration, respectively, presented as median (min-max). Panels (**B**) and (**F**) show erythrocyte DPPH scavenging activity and plasma ORAC values, respectively, presented as mean ± standard deviation. Units are expressed as percentage of scavenging (% scavenging); units per milliliter of plasma (U/mL); nanomoles per milliliter of plasma (nmol/mL), and micromoles of Trolox equivalent (µM TE). Note: Antioxidant-related markers are reported as descriptive indicators of cohort-level biochemical status; standardized clinical reference ranges for the third trimester of pregnancy are not available.

To evaluate the association between VD status and antioxidant/redox-related markers, glutathione-related markers and total antioxidant capacity assays were compared between groups (Figure 2). No significant differences were observed in plasma ORAC values or erythrocyte DPPH radical scavenging capacity (*p* > 0.05 for both).

In contrast, several glutathione-related markers differed between groups. Women with VDI exhibited lower GSH concentrations (median: 0.66 vs. 0.97 nmol/mL); *p* = 0.002), reduced GST activity (median 0.020 vs. 0.024 U/mL); *p* = 0.001), and slightly lower GPx activity (median 0.045 vs. 0.048 U/mL; *p* = 0.047). In addition, plasma DPPH scavenging capacity was significantly lower in women with VDI (30.65 ± 8.63% vs. 34.39 ± 11.13%; *p*= 0.04).

Overall, these findings indicate that VDI was associated with lower values of selected glutathione-related antioxidant markers, without implying functional impairment or causal effects.

### 3.3. Correlations Between VD Concentrations and Measured Markers

**Figure 3:**
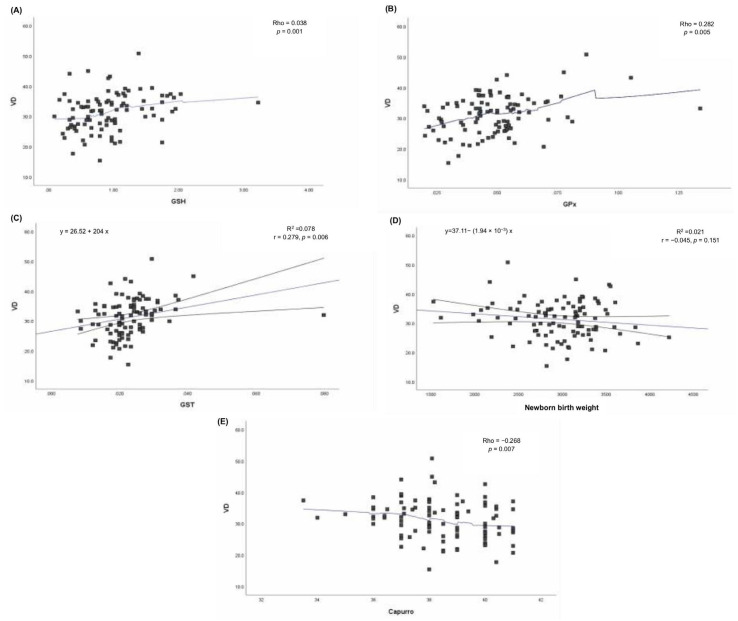
Bivariate correlation between maternal serum 25-OHD concentrations and selected antioxidant markers and neonatal outcomes. Scatter plots illustrate the association between VD levels and (**A**) glutathione (GSH), (**B**) glutathione peroxidase (GPx), (**C**) glutathione S-transferase (GST), (**D**) newborn birth weight, and (**E**) gestational age estimated by the Capurro score. Solid lines represent fitted regression lines with 95% confidence intervals. Pearson’s or Spearman’s coefficients are reported as appropriate.

Correlations analyses were performed to further characterize associations between VD concentrations and antioxidant-related markers, using Pearson’s (r) or Spearman’s (Rho) correlation coefficient as appropriate. Serum VD concentrations were positively correlated with GSH concentrations (Rho = 0.038, *p* = 0.001), GPx (Rho = 0.282, *p* = 0.005), and GST (r = 0.279, *p* = 0.006) (Figure 3A–C), indicating that higher VD concentrations were associated with higher values of these antioxidant-related markers.

No significant association was observed between VD concentrations and newborn birth weight (r = −0.045, *p* = 0.151; Figure 3D). In contrast, a modest negative correlation was observed between VD concentration and gestational age estimated by the Capurro score (Rho = −0.268, *p* = 0.007; Figure 3E). This association should be interpreted cautiously given the high prevalence of cesarean delivery in the cohort and the cross-sectional design of the study.

### 3.4. Maternal, Newborn, and Metabolic Characteristics by VD Status

To explore potential clinical and metabolic differences according to VD status, maternal and neonatal characteristics were compared between women with VD sufficiency, and those with VDI (Table 1). Most demographic, anthropometric, and obstetric variables were comparable between groups. However, women classified as VDI reported a significantly higher prevalence of secondhand tobacco smoke exposure compared with women with sufficient VD concentrations (47.6% vs. 22.8%, *p* = 0.017). No significant differences were observed in age, BMI category, gestational weight gain, education level, or obstetric outcomes such as GDM or preeclampsia (all *p* > 0.05).

Regarding neonatal outcomes, infants born to mothers with VDI had a higher mean birth weight (3083.8 ± 406.4 vs. 2864.8 ± 491.1, *p* = 0.021), and slightly higher gestational age estimated by the Capurro score (median: 39 vs. 38 weeks, *p* = 0.003). We examined the variability of newborn weight using a multivariate linear regression model that included BMI, maternal weight gain, delivery resolution, infant height, Apgar score, admission to the NICU, and VD values. The only factors associated with the observed variation in neonatal weight were newborn height and maternal weight gain (Supplementary Material, Table S3). No differences were observed in other neonatal anthropometric or clinical parameters.

Maternal biochemical and metabolic profile was also examined (Table 2). As expected, stratification by VD status resulted in significantly different serum 25-OHD concentrations (35.67 ± 3.90 ng/mL vs. 25.53 ± 3.40 ng/mL; *p* < 0.001). In contrast, no significant differences were observed in fasting glucose, leukocyte count, lipid profile (triglycerides, total cholesterol, LDL-C, and HDL-C), uric acid, or hemoglobin levels (all *p* > 0.05). These findings indicate that, in this cohort, VDI was not associated with differences in routine metabolic or hematological markers.

### 3.5. Influence of Season of Sampling on Maternal VD Status

Given the well-documented influence of environmental factors on serum VD concentrations, the distribution on maternal VD status across seasons was examined (Table 3). A significant association between season of sampling and VD status was observed (*p* < 0.001).

VDI was most frequently observed among women sampled during spring where 66.7% were classified as VDI. During summer, VD status was more evenly distributed, with 52.9% of participants classified as sufficient. In contrast, a higher proportion of women sampled during autumn (85.7%) and winter (93.8%) were classified as VD-sufficient.

Overall, these findings indicate a seasonal variation in maternal VD status within this cohort.

### 3.6. Multivariate Predictors of VD Concentrations

Multiple linear regression analyses were conducted to examine factors independently associated with maternal VD concentrations (Table 4). In an initial model including OS markers and adjusted for maternal age and BMI, GST and GPx were independently associated with VD concentrations, jointly explaining 18.4% of the variability.

Variables that demonstrated relevance in bivariate analysis were incorporated into the second model. The season of the year in which the sample was collected was found to be the best predictor of VD variability, and it was also found to be somewhat inversely correlated with the Capurro score. Together, these variables explained 41% of the variability in VD concentrations. Season of sampling showed the strongest association. After adjustment for these covariates, OS markers were no longer independently associated with VD concentrations.

## 4. Discussion

Our study identifies significant associations between maternal VDI and selected environmental factors, neonatal outcomes, and maternal redox-related biochemical parameters. First, when examining factors associated with VDI, we observed that women exposed to secondhand smoke exhibited a significantly higher prevalence of VDI compared with non-exposed women. This finding is consistent with previous evidence linking passive smoking to lower circulation VD concentrations during pregnancy [42,43]. Experimental and mechanistic studies suggest that tobacco-derived toxicants may interfere with VD synthesis or metabolism, including alterations in enzymes involved in VD activation or receptor signaling [44,45]. However, because such pathways were not evaluated in the present study, it is not possible to determine whether these mechanisms contributed to the associations observed in this cohort.

Beyond environmental exposures, our results provide an observational perspective on the relationship between maternal VDI and fetal growth. Unexpectedly, women with VDI delivered neonates with significantly higher birth weight compared with those classified as VD-sufficient, in the absence of over deficiency, whereas low birth weight and intrauterine growth restriction are more consistently reported in the literature [46,47]. This finding may therefore reflect population-specific characteristics rather than a direct biological effect of maternal VD status. Supporting this interpretation, previous cohorts with comparable non-deficient VD concentrations have reported heterogeneous results, ranging from null to positive associations with birth weight [48,49,50]. Consistent with this, correlation analyses revealed a weak and non-significant association between maternal VD concentrations and birth weight, suggesting that within the insufficiency range, VD is unlikely to be a dominant determinant of neonatal weight. Other contextual factors, including overall nutritional adequacy or socioeconomic conditions, may have contributed to favorable fetal growth and attenuated subtle associations.

An additional unexpected finding was the association between maternal VDI and longer gestational age as estimated by the Capurro score. Although a statistically significant negative correlation between VD concentrations and gestational age was observed, this result must be interpreted with caution. The very high prevalence of cesarean delivery in this cohort (>80%) represents an important contextual factor when interpreting neonatal outcomes. While birth weight is generally considered as a robust anthropometric measure, mode of delivery may influence immediate neonatal physiological status and early postnatal measurements. Moreover, the predominance of scheduled cesarean sections likely reflects clinical decision-making rather than spontaneous labor onset, potentially introducing systematic differences in gestational age estimation and neonatal maturity. Consequently, both the observed associations with birth weight and the correlations involving gestational age may partly reflect obstetric practices rather than underlying biological relationships with maternal VD status.

Environmental and demographic determinants play a central role in VD availability during pregnancy, with seasonality representing a key factor. Cutaneous synthesis driven by ultraviolet B (UVB) exposure (290–315 nm) constitutes the primary source of VD, although its efficiency is modulated by age, skin pigmentation, UVB intensity, and behavioral factors such as time spent outdoors [44]. In our cohort, an unexpected seasonal pattern was observed, with the highest prevalence of VDI occurring during spring and higher rates of sufficiency during autumn and winter. Given Mexico City’s high altitude (2.240 m), where ambient UVB radiation remains relatively elevated throughout the year [51,52,53,54], this pattern is unlikely to be explained solely by environmental UVB availability. In the absence of direct measurements of sun exposure, behavioral factors-such as seasonal avoidance of outdoor activity during warmer months or increased outdoor exposure during milder seasons-may have contributed to this pattern. However, without objective exposure data, this interpretation remains speculative.

In this context, urban and behavioral factors may further contribute to seasonal variability in VD status. Urban mobility restrictions, such as the “Hoy no circula” program in Mexico City, which limits vehicular use on specific days, may indirectly influence daily routines, transportation choices, and time spent outdoors. Although speculative, this interpretation aligns with previous studies showing that culture and climatic factors can invert expected seasonal patterns of VD synthesis [55,56,57,58,59]. Nonetheless, causal inferences cannot be drawn from these observations.

Regarding maternal biochemical parameters, VDI was not associated with differences in standard metabolic markers, including glucose, lipid profile, uric acid, leukocyte count, and hemoglobin levels. These findings suggest that, in this cohort, VDI was not accompanied by measurable alterations in routine biochemical indicators. This observation is consistent with intervention studies reporting limited metabolic effects of VD supplementation beyond changes in serum VD concentrations [60], although discrepancies across studies may reflect differences in baseline status, gestational timing, or unmeasured dietary and genetic factors [61].

A distinctive aspect of our study is the evaluation of maternal redox-related parameters. Women with VDI exhibited lower activities of GSH, GST, and GPx compared with those with sufficient VD concentrations. Importantly, these assays are not validated clinical biomarkers, are sensitive to pre-analytical variability, and lack established reference ranges for pregnancy. Accordingly, they should be interpreted as exploratory indicators of redox-related biochemical status rather than measures of systemic antioxidant capacity or OS. While previous studies have reported associations between VD status and components of antioxidant pathways [30,62], and pregnancy is characterized by a physiologically increased oxidative state-whose dysregulation has been linked to complications such as preeclampsia, DMG and preterm birth [25,28].

Experimental and supplementation studies have proposed interactions between 1,25(OH)_2_D_3_ and glutathione-related enzymes [26,63,64], with associations reported for GPx and GST activity [65,66,67,68,69,70,71]. The enzymatic pattern observed in our cohort is compatible with, but does not confirm, the involvement of regulatory pathways described in the literature, such as redox-sensitive transcriptional responses [72,73]. These references are provided solely as contextual background and should not be interpreted as evidence of mechanistic involvement in this study.

Finally, multivariate analysis places these findings within a broader explanatory framework. Redox-related parameters accounted for a modest proportion (18.4%) of the variability in maternal VD concentrations, whereas seasonality, and neonatal maturity (Capurro score), explained for the majority (41%). This underscores that environmental and demographic factors are more strongly associated with maternal VD status than redox-related measures and reinforces the need for cautious interpretation of exploratory biochemical associations in observational pregnancy studies.

## 5. Conclusions

Our findings indicate that VDI during gestation is associated with environmental factors, including secondhand tobacco smoke exposure and season of the year, as well as sociodemographic characteristics such as maternal age. Although lower activities of selected redox-related enzymes (GSH, GST, and GPx) were observed in women with VDI, these measures should be interpreted as exploratory biochemical correlates rather than as validated indicators of antioxidant capacity or clinical OS. Within the multifactorial framework that regulates VD status during pregnancy, redox-related parameters appear to make a modest contribution compared with environmental determinants.

*Limitations and future research:* Several limitations should be considered when interpreting our results. The cross-sectional design precludes the establishment of directionality or causality between VDI and the observed associations. Exposure tobacco smoke was assessed through self-report, which may be subject to recall or reporting bias. In addition, the high rate of cesarean deliveries limits the interpretability of gestational age, as this variable may have been influenced by clinical decision-making rather than physiological timing.

Although key components of the antioxidant system were evaluated, other relevant enzymatic defenses (e.g., superoxide dismutase, catalase) and markers of oxidative damage (e.g., MDA), were not included, limiting the scope of redox characterization. Furthermore, sunlight exposure, outdoor physical activity, and dietary intake were not directly assessed. In particular, occupational sun exposure was classified using self-report categories (high, medium, low), a subjective approach that lacks the precision of objective exposure measures. This limitation restricts accurate quantification of individual sun exposure and ability to disentangle its contribution to VD status. Consequently, while an unexpected seasonal pattern in VDI prevalence was observed, the absence of objective UVB intensity data or direct personal sun exposure metrics precludes definitive conclusions regarding the behavioral determinants proposed.

Future research should prioritize longitudinal designs to clarify the temporal relationships between maternal VD status and redox-related biochemical changes during pregnancy. Incorporating objective measures of tobacco exposure (e.g., salivary or blood cotinine), expanding the panel of OS and antioxidant markers, and integrating objective assessment of sun exposure strengthen future analyses. Finally, mechanistic studies using experimental models will be necessary to determine whether and how VD may interact with antioxidant pathways within the maternal–fetal environment.

## Figures and Tables

**Table 1:** Maternal, obstetric, and neonatal characteristics stratified by maternal VD status.

Characteristic	Sufficiency (*n* = 57)	Insufficiency (*n* = 42)	*p*-Value
Maternal data			
Age, years	30.1 ± 4.4	29.8 ± 4.7	0.780
Weight, kg	65 (45–100)	64.5 (45–106)	0.363
Height, cm	160 (150–175)	159 (142–170)	0.504
BMI category, n (%)			
Normal	18 (31.6)	20 (47.6)	0.256
Overweight	25 (43.9)	15 (35.7)	
Obese	14 (24.6)	7 (16.7)	
Weight gain, kg	9.5 ± 4.7	10.1 ± 5.5	0.541
Smoking status, n (%)	13 (22.8)	20 (47.6)	**0.010**
Education, n (%)			
Secondary education	3 (5.3)	4 (9.5)	0.755
High school	17 (29.8)	14 (33.3)	
Bachelor’s degree	36 (63.2)	24 (57.1)	
Postgraduate	1 (1.8)	0 (0.0)	
Occupational sun exposure, n (%)			
High exposure	5 (8.8)	1 (2.4)	0.504
Medium exposure	39 (68.4)	31 (73.8)	
Low exposure	13 (22.8)	10 (23.8)	
VD supplementation (400–500 Ul), n (%)	26 (45.6)	20 (47.6)	0.843
GDM, n (%)	3 (5.3)	1 (2.4)	0.635
Preeclampsia, n (%)	3 (5.3)	1 (2.4)	0.635
Abortions, n	0 (0–2)	0 (0–3)	0.240
PPB (<37 weeks), n	0 (0–1)	0 (0–1)	0.681
PFB (≥37 weeks), n	1 (0–3)	1 (0–4)	0.681
Delivery mode, n (%)			
Cesarean	47 (82.5)	35 (83.3)	0.909
Vaginal delivery	10 (17.5)	7 (16.7)	
Newborn outcomes			
Weight, g	2864.8 ± 491.1	3083.8 ± 406.4	**0.021**
Height, cm	49 (40–54)	49 (44–53)	0.308
APGAR, score	8 (5–9)	8 (5–9)	0.257
Capurro score, weeks	38 (34–41)	39 (36–41)	**0.003**
Head circumference, cm	34 (29–36)	34 (32–36)	0.099
Mid-upper arm, cm	11 (8–12)	11 (9–14)	0.466

Data are presented as mean ± standard deviation, median (min–max), or n (%). A *p*-value in black denotes statistical significance. BMI: body mass index; GDM: gestational diabetes mellitus; VD: vitamin D; PPB: previous preterm births; PFP: previous full-term births.

**Table 2:** Maternal biochemical and metabolic parameters according to VD status.

Variable	Sufficiency (*n* = 57)	Insufficiency (*n* = 42)	*p*-Value
Vitamin D (ng/mL)	35.6 ± 3.90	25.5 ± 3.40	**0.000**
Glucose (mg/dL)	82.5 (61–258)	78.0 (62–200)	0.763
Leukocytes (×10^9^/L)	9.29 (5.0–17.2)	8.29 (5.46–18.6)	0.241
Triglycerides (mg/dL)	193.5 (118–362)	201.0 (103–513)	0.709
Cholesterol (mg/dL)	196.1 (106–267)	193.0 (136–274)	0.367
LDL-C (mg/dL)	97.5 (33–169)	85 (54–149)	0.110
HDL-C (mg/dL)	55.9 ± 12.36	56.4 ± 15.09	0.878
Uric acid (mg/dL)	4.54 (3–8)	4.80 (3–8)	0.373
Hemoglobin (g/dL)	13.0 (8.8–15.5)	12.9 (10.7–15.4)	0.318

Data presented as mean ± standard deviation or median (range). A *p*-value in black denotes statistical significance *p* < 0.001. LDL-C: low-density lipoprotein cholesterol; HDL-C: high-density lipoprotein cholesterol.

**Table 3:** Distribution of maternal VD status according to season of sample collection.

Season	Sufficiency, *n* (%)	Insufficiency, *n* (%)	*p*-Value
Spring	15 (33.3)	30 (66.7)	**<0.001**
Summer	9 (52.9)	8 (47.1)	
Autumn	18 (85.7)	3 (14.3)	
Winter	15 (93.8)	1 (6.3)	

Data presented as number of participants and percentage (n %). A *p*-value in black denotes statistical significance of the overall seasonal variation (Chi-square test).

**Table 4:** Multiple linear regression models identifying independent predictors of maternal VD concentrations.

Variable	Coefficient (S.E)	β	*p*-Value	R^2^, *p*-Value
GST ^a^	264.95 (95.9)	0.291	0.007	0.184, <0.0001
GPx ^a^	86.4 (32.6)	0.279	0.010	
Season ^b^	3.35 (0.448)	0.606	<0.001	0.41, <0.0001
Capurro ^b^	−0.765 (0.304)	−0.204	0.014	

^a^ Model adjusted by BMI and age. Excluded variables in the stepwise model: DPPH in plasma and erythrocytes, ORAC, and GSH. Radical 2,2-diphenyl-2-2picrylhydrazyl (DPPH); glutathione reduced (GSH); glutathione S-transferase (GST); glutathione peroxidase (GPx); oxygen radical absorbance capacity (ORAC). ^b^ The model included GPx, GST, GSH, infant weight, Capurro index, mother’s smoking status, and season of sampling.

## Data Availability

The original data presented in this study are openly available and included in the article/Supplementary Material.

## Supplementary Materials

The following supporting information can be downloaded at https://www.mdpi.com/article/10.3390/metabo16010019/s1, Table S1: Variables description; Table S2: Validation of Multivariate linear regression model’s; Table S3: Linear regression of newborn’s weight.

## Abbreviations

The following abbreviations are used in this manuscript:

1,25(OH)_2_D_3_	1,25-dihydroxivitamin D3
25OHD	25-hydroxivitamin D
Apgar	Apgar score
BMI	Body mass index
Capurro	Capurro score
CI	Confidence intervals
DPPH	Radical 2,2-diphenyl-2-2picrylhydrazyl
ES	Endocrine society
GDM	Gestational diabetes mellitus
GPx	Glutathione peroxidase
GSH	Glutathione reduced
GSSG	Oxidized glutathione
GST	Glutathione-S-transferase
HDL-C	High-density lipoprotein cholesterol
IOM	Institute of medicine
IQR	Interquartile range
LDL-C	Low-density lipoprotein cholesterol
MDA	Malondialdehyde
NADPH	Nicotinamide Adenine Dinucleotide Phosphate
ORAC	Oxygen Radical Absorbance Capacity
OS	Oxidative stress
ROS	Reactive oxygen species
UVB	Ultraviolet B radiation
VD	Vitamin D
VDD	Vitamin D deficiency
VDI	Vitamin D insufficiency
VDR	Vitamin D receptor
SZA	Solar Zenith Angle
