# Supplementary material for: Maternal Vitamin D Status, Oxidative Stress, and Implications for Neonatal Development: A Cross-Sectional Study
Source: Metabolites. 2025 Dec 24;16(1):19. doi: 10.3390/metabo16010019 (PMC12843661; doi:10.3390/metabo16010019)
Supplement: Supplementary file 1 [file metabolites-16-00019-s001.zip › metabolites-4035332-supplementary.pdf]

Supplementary material

Table S1. Variables description

Exposure variables			
**Variable**	**Operational definition**	**Type**	**Measurement Method**
Vitamin D status	- Serum 25-hydroxyvitamin D [25(OH)D] concentration (ng/mL) Deficiency (<20 ng/mL), insufficiency (21-29 ng/mL), sufficiency (30-50 ng/mL) (Endocrine Society Guidelines)	Quantitative	Liquid Chromatography-Tandem Mass Spectrometry (LC-MS/MS)
Antioxidant statues	- DPPH: Free radical scavenging activity (% inhibition)<br>- ORAC: Oxygen radical absorbance capacity (μM TE/g)<br>- GSH: Intracellular concentration of reduced glutathione (μM/mg protein or nmol/g plasma)<br>- GPx: Glutathione peroxidase activity (U/mL)<br>- GST: Glutathione S-transferase activity (U/mL)	Quantitative	Spectrophotometry (UV/VIS)
Supplementation	- Use of prenatal supplements (vitamins, iron, etc.)<br>yes/no	Qualitative	Structured interview
Smoking status	- Active: ≥ cigarette/day during pregnancy<br>- Pasive: secondhand exposure ≥ 1h/day at home	Qualitative	Validated questionnaire

Outcome variables: Maternal outcomes		
**Variable**	**Operational definition**	**Type**
Delivery mode	- Vaginal delivery<br>- Cesarean section (elective/emergency)	Qualitative
Obstetric complications	- Gestational diabetes: The International Association of the Diabetes and Pregnancy Study Groups (IADPSG) criteria.<br>- Preeclampsia/eclampsia: The American College of Obstetrician and Gynecologists (ACOG) criteria (Bloom pressure ≥ 140/90 mmHg + proteinuria).<br>- Miscarriages: <20 weeks of gestation.<br>- Premature birth: <37 weeks of gestation.	Qualitative

Neonatal outcomes		
**Variable**	**Operational definition**	**Type**
APGAR score	- Assessed at 5 minutes (scale 0-10)	Quantitative
Capurro score	- Postnatal gestation age estimation via physical examination	Quantitative

Birth weight/length	- Measured within the first postnatal hour (g/cm)	Quantitative
NICU admission	- Requirement of neonatal intensive care (yes/no)	Qualitative
**Confounding variables**		
**Variable**	**Operational definition**	**Type**
Pre-pregnancy	- Weight (kg)/height^2 (m^2) before gestation	Quantitative
Maternal age	- Years at delivery	Quantitative
Education level	- Primary<br>- Secondary<br>- University<br>- Postgraduate	Qualitative
Gestational weight gain	- Difference between final weigth and pre-pregnancy BMI (kg)<br>Underweight (<18.5), normal (18.5 - 24.9), overweight (25-29.9), obese (>30) (WHO criteria)	Quantitative
Obstetric history	- Term/preterm previous pregnancies<br>- Miscarriages (spontaneous/induced)	Quantitative / qualitative
Occupational sun exposure	Derived from self-reported occupation and classified by researcher consensus based on the estimated percentage of the workday spent outdoors:<br>- High: >75% outdoors<br>- Medium: 25 – 75% outdoors, or frequent intermittent exposure<br>- Low: <25% outdoors	Qualitative
Season at blood sampling	The climatic period of the year at the time of blood draw, considered key potential confounder for 25OHD levels due to variations in sunlight exposure.<br>Spring: from March 20 to June 20<br>Summer: from June 21 to September 21<br>Autumn: from September 22 to December 20<br>Winter: from December 21 to March 19	Qualitative
**Biochemical variables (Covariables)**		
**Variable**	**Operational definition**	**Type**
Glucose	- Serum glucose (mg/dL)	Quantitative
Lipid profile	- Total cholesterol, HDL, LDL (mg/dL)<br>- Triglycerides (mg/dL)	Quantitative
Uric acid	- Serum concentration (mg/dL)	Quantitative
Hemoglobin	- Venous blood concentration (g/dL)	Quantitative

Table S2. Validation of Multivariate linear regression model's

Model 1:		
VIF	GSH	1.19
	GST	1.18
	GPX	1.08
	DPPH_plasma	1.17
	DPPH_erytrochytes	1.05
	ORAC	1.03
	BMI	1.05
	Age	1.10
Skewness/Kurtosis tests for Normality	Residuals	Chi2.4.4, P=0.1109
Shapiro-Wilk W test for normal data	Residuals	Z=0.52, P=0.300
White's test for		Chi2, P
Ho: homoskedasticity against	Heteroskedasticity |	38.76, 0.6952
Ha: unrestricted	Skewness |	3.04, 0.9316
heteroskedasticity	Kurtosis |	4.52, 0.0335
Durbin-Watson	1.329	
Model 2:		
VIF	Season	1.87
	Capurro	1.82
	Newborn Weight	1.76
	GSH	1.34
	GPx	1.31
	GST	1.19
	Smooking	1.11
Skewness/Kurtosis tests for Normality	Residuals	Chi2. 0.11 P=0.9449
Shapiro-Wilk W test for normal data	Residuals	Z=-2.63, P=0.995
White's test for		Chi2, P
Ho: homoskedasticity against	Heteroskedasticity |	38.9, 0.2584
Ha: unrestricted	Skewness |	12.16, 0.0953
heteroskedasticity	Kurtosis |	0.26.52, 0.6092
Durbin-Watson	1.509	

Table S3. Linear regression of newborn's weight

Variable	Coefficient (S.E)	β	p-value	R^2, p-value
Height	159.24 (13.5)	0.757	<0.001	0.61, <0.0001
Mother's weight gain	12.2 (6.1)	0128	0.048	

Stepwise model including newborn's weight and BMI, maternal weight gain, delivery resolution, infant height, Apgar score, admission to NICU, and VD values.
All VIF values were <2 and residuals had normal distribution.
